# Supplementary material for: Mendelian Randomization Study of Lipid Metabolites Reveals Causal Associations with Heel Bone Mineral Density
Source: Nutrients. 2023 Sep 27;15(19):4160. doi: 10.3390/nu15194160 (PMC10574167; doi:10.3390/nu15194160)
Supplement: Supplementary file 1 [file nutrients-15-04160-s001.zip › Supplementary Figures.pdf]

Supplementary Figures

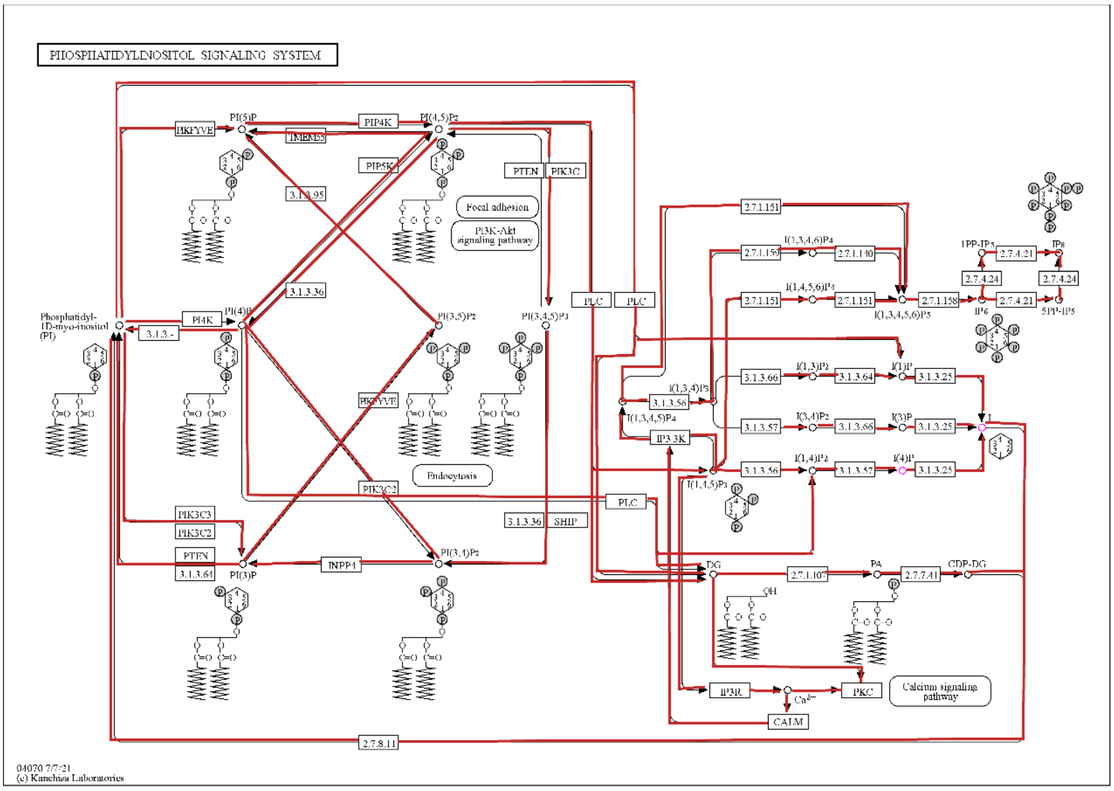

Supplementary Figure S1. Downstream metabolic molecules of myo-inositol in the phosphatidylinositol signaling system. We obtained the photoshatidylinositol signaling system pathway map related to myo-inositol from the KEGG database and marked the downstream reactions of myo-inositol with red boxes and lines. Subsequently, we obtained SNPs for downstream metabolites in the IEU OpenGWAS project and explored their causal relationship with H-BMD. URL of the IEU OpenGWAS project: https://gwas.mrcieu.ac.uk/ (accessed on 31 August 2023).

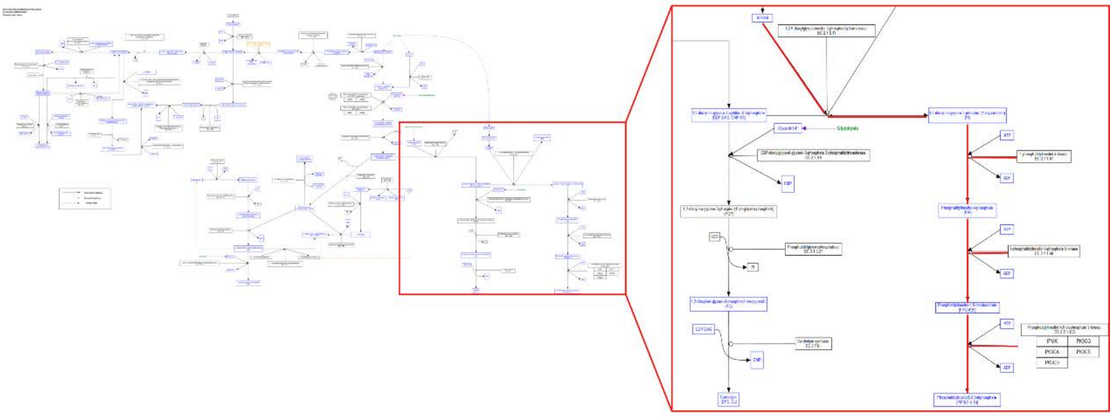

Supplementary Figure S2. Downstream molecules of myo-inositol in the glycerophospholipid biosynthetic pathway. We obtained metabolic pathways containing myo-inositol or inositol and corresponding figure in the Wikipathway database, and marked the downstream reactions of myo-inositol with red boxes and lines. Subsequently, we obtained SNPs for downstream metabolites in the IEU OpenGWAS project and explored their causal relationship with H-BMD. URL of the IEU OpenGWAS project: https://gwas.mrcieu.ac.uk/ (accessed on 31 August 2023).

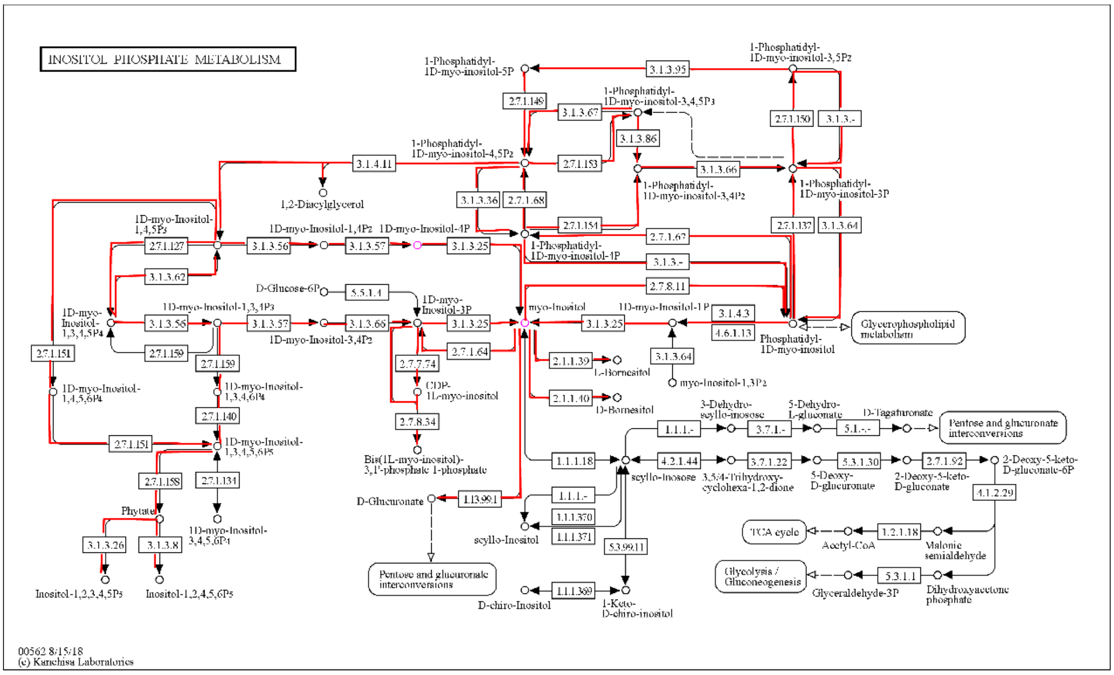

Supplementary Figure S3. Downstream molecules of myo-inositol in the inositol phosphate metabolism. We obtained the phosphate metabolism pathway map related to myo-inositol from the KEGG database and marked the downstream reactions of myo-inositol with red boxes and lines. Subsequently, we obtained SNPs for downstream metabolites in the IEU OpenGWAS project and explored their causal relationship with H-BMD. URL of the IEU OpenGWAS project: https://gwas.mrcieu.ac.uk/ (accessed on 31 August 2023).

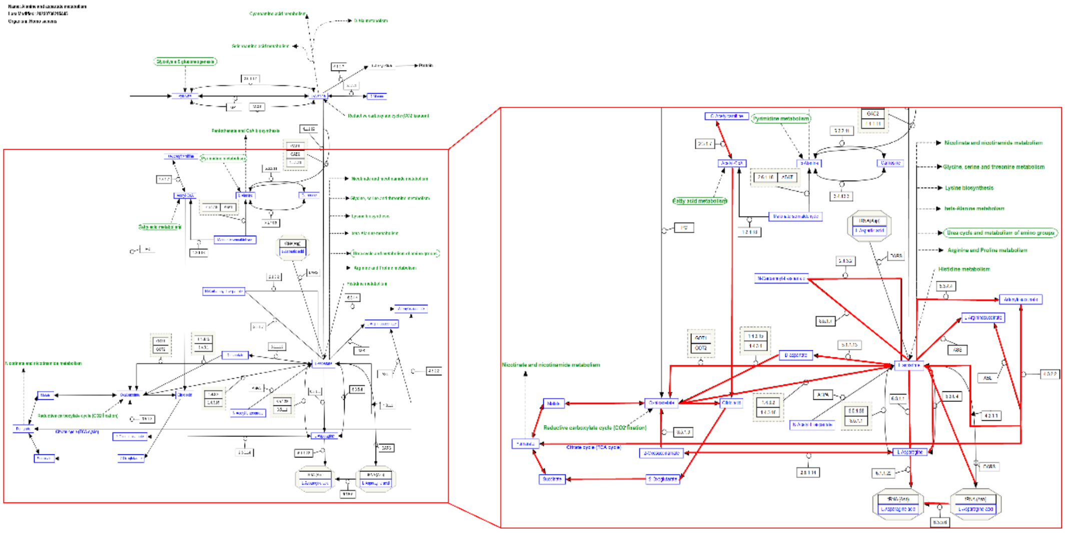

Supplementary Figure S4. Downstream molecules of acetylcarnitine in the alanine and aspartate metabolism pathway. We obtained metabolic pathways containing acetylcarnitine and corresponding figure in the Wikipathway database, and marked the downstream reactions of acetylcarnitine with red boxes and lines. Subsequently, we obtained SNPs for downstream metabolites in the IEU OpenGWAS project and explored their causal relationship with H-BMD. URL of the IEU OpenGWAS project: https://gwas.mrcieu.ac.uk/ (accessed on 31 August 2023).

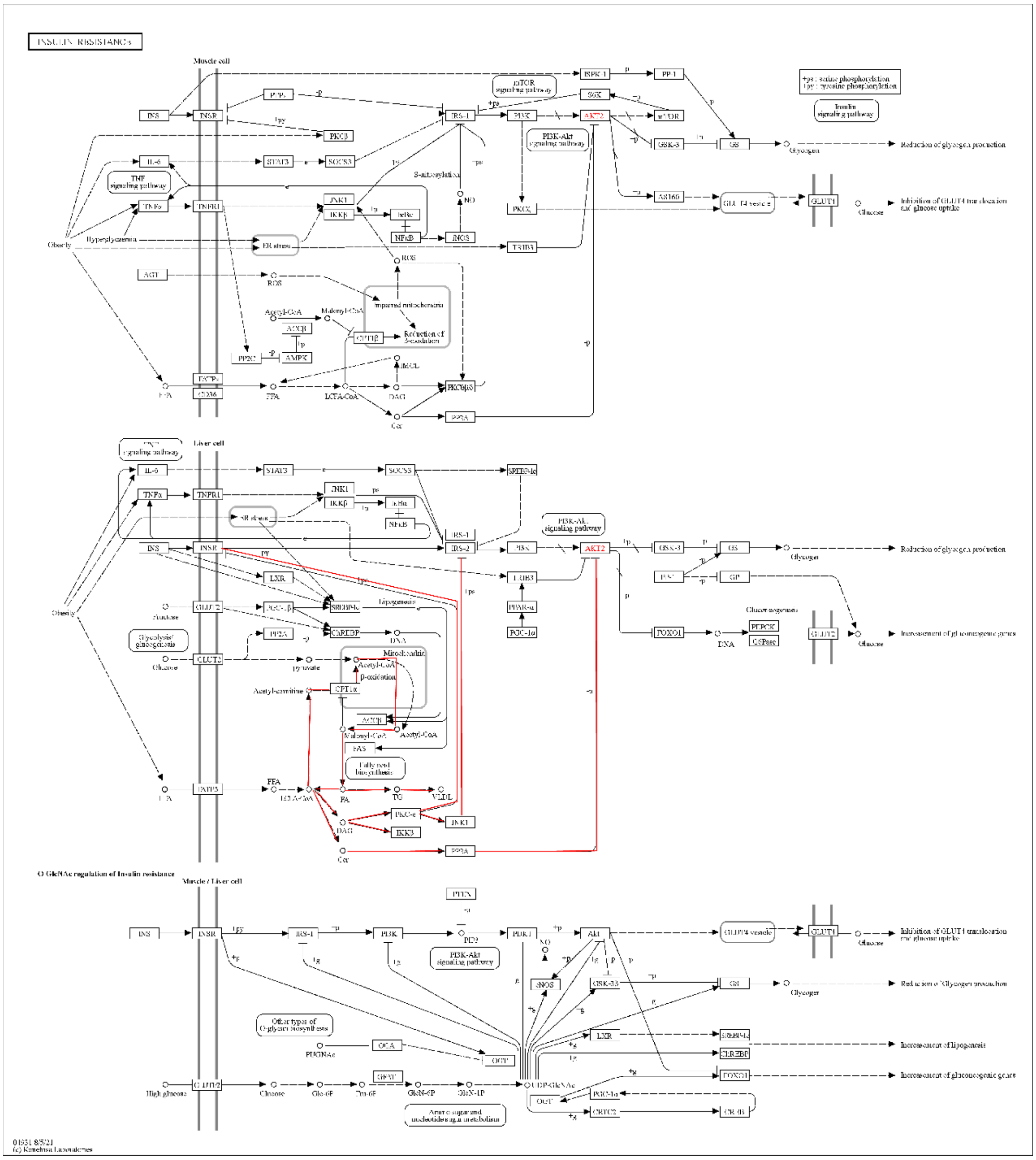

Supplementary Figure S5. Downstream molecules of acetylcarnitine in the insulin resistance pathway. We obtained metabolic pathways containing acetylcarnitine and corresponding figure in the Wikipathway database, and marked the downstream reactions of acetylcarnitine with red boxes and lines. Subsequently, we obtained SNPs for downstream metabolites in the IEU OpenGWAS project and explored their causal relationship with H-BMD. URL of the IEU OpenGWAS project: https://gwas.mrcieu.ac.uk/ (accessed on 31 August 2023).
