# Supplementary material for: Mendelian Randomization Study of Lipid Metabolites Reveals Causal Associations with Heel Bone Mineral Density
Source: Nutrients. 2023 Sep 27;15(19):4160. doi: 10.3390/nu15194160 (PMC10574167; doi:10.3390/nu15194160)
Supplement: Supplementary file 1 [file nutrients-15-04160-s001.zip › Supplementary Tables.pdf]

Supplementary Tables

Table S1. The 130 lipid metabolites selected in this study and their corresponding codes in IEU OpenGWAS project. These metabolites belong to lipids on the super pathway. Code: The code corresponding to the metabolites listed in the IEU OpenGWAS project. URL of the IEU OpenGWAS project: https://gwas.mrcieu.ac.uk/ (accessed on 31 August 2023).

Metabolite	Status	Pathway	Code
Cholate	Known	Bile acid metabolism	met-a-438
Deoxycholate	Known	Bile acid metabolism	met-a-320
Glycochenodeoxycholate	Known	Bile acid metabolism	met-a-470
Glycocholate	Known	Bile acid metabolism	met-a-404
Glycodeoxycholate	Known	Bile acid metabolism	met-a-405
Glycoursodeoxycholate	Known	Bile acid metabolism	#N/A
Hyodeoxycholate	Known	Bile acid metabolism	met-a-447
Taurochenodeoxycholate	Known	Bile acid metabolism	met-a-406
Taurocholate	Known	Bile acid metabolism	met-a-407
Taurodeoxycholate	Known	Bile acid metabolism	met-a-369
Taurolithocholate 3-sulfate	Known	Bile acid metabolism	met-a-740
Ursodeoxycholate	Known	Bile acid metabolism	met-a-346
2-tetradecenoyl carnitine	Known	Carnitine metabolism	met-a-702
3-dehydrocarnitine	Known	Carnitine metabolism	met-a-500
Acetylcarnitine	Known	Carnitine metabolism	met-a-463
Carnitine	Known	Carnitine metabolism	met-a-379
Cis-4-decenoyl carnitine	Known	Carnitine metabolism	met-a-753
Decanoylcarnitine	Known	Carnitine metabolism	met-a-618

Hexanoylcarnitine	Known	Carnitine metabolism	met-a-467
Laurylcarnitine	Known	Carnitine metabolism	met-a-668
Octanoylcarnitine	Known	Carnitine metabolism	met-a-615
Oleoylcarnitine	Known	Carnitine metabolism	met-a-681
Palmitoylcarnitine	Known	Carnitine metabolism	met-a-434
Stearoylcarnitine	Known	Carnitine metabolism	met-a-653
Dihomo-linolenate (20:3n3 or n6)	Known	Essential fatty acid	met-a-712
Docosahexaenoate (DHA; 22:6n3)	Known	Essential fatty acid	met-a-409
Docosapentaenoate (n3 DPA; 22:5n3)	Known	Essential fatty acid	met-a-483
Eicosapentaenoate (EPA; 20:5n3)	Known	Essential fatty acid	met-a-402
Linoleate (18:2n6)	Known	Essential fatty acid	met-a-317
Linolenate [alpha or gamma; (18:3n3 or 6)]	Known	Essential fatty acid	met-a-628
Isovalerate	Known	Fatty acid metabolism	met-a-672
Butyrylcarnitine	Known	Fatty acid metabolism (also BCAA metabolism)	met-a-476
Propionylcarnitine	Known	Fatty acid metabolism (also BCAA metabolism)	met-a-479
Linoleamide (18:2n6)	Known	Fatty acid, amide	#N/A
Oleamide	Known	Fatty acid, amide	#N/A
15-methylpalmitate (isobar with 2-methylpalmitate)	Known	Fatty acid, branched	met-a-754
2-hydroxyglutarate	Known	Fatty acid, dicarboxylate	met-a-749
3-carboxy-4-methyl-5-propyl-2-furanpropanoate (CMPF)	Known	Fatty acid, dicarboxylate	met-a-461
Dodecanedioate	Known	Fatty acid, dicarboxylate	met-a-473
Hexadecanedioate	Known	Fatty acid, dicarboxylate	met-a-711
Octadecanedioate	Known	Fatty acid, dicarboxylate	met-a-735
Sebacate (decanedioate)	Known	Fatty acid, dicarboxylate	#N/A
Tetradecanedioate	Known	Fatty acid, dicarboxylate	met-a-709
N-Butyl Oleate	Known	Fatty acid, ester	met-a-738

2-hydroxypalmitate	Known	Fatty acid, monohydroxy	met-a-710
2-hydroxystearate	Known	Fatty acid, monohydroxy	met-a-397
Choline	Known	Glycerolipid metabolism	met-a-380
Glycerol	Known	Glycerolipid metabolism	met-a-374
Glycerol 3-phosphate (G3P)	Known	Glycerolipid metabolism	met-a-377
Glycerophosphorylcholine (GPC)	Known	Glycerolipid metabolism	met-a-387
Chiro-inositol	Known	Inositol metabolism	met-a-745
Inositol 1-phosphate (I1P)	Known	Inositol metabolism	#N/A
Myo-inositol	linoleat	Inositol metabolism	met-a-417
Scyllo-inositol	Known	Inositol metabolism	met-a-472
3-hydroxybutyrate (BHBA)	Known	Ketone bodies	met-a-311
10-heptadecenoate (17:1n7)	Known	Long chain fatty acid	met-a-625
10-nonadecenoate (19:1n9)	Known	Long chain fatty acid	met-a-626
Adrenate (22:4n6)	Known	Long chain fatty acid	met-a-537
Arachidonate (20:4n6)	Known	Long chain fatty acid	met-a-319
Dihomo-linoleate (20:2n6)	Known	Long chain fatty acid	met-a-395
Eicosenoate (20:1n9 or 11)	Known	Long chain fatty acid	met-a-586
Margarate (17:0)	Known	Long chain fatty acid	met-a-321
Myristate (14:0)	Known	Long chain fatty acid	met-a-334
Myristoleate (14:1n5)	Known	Long chain fatty acid	met-a-477
Nonadecanoate (19:0)	Known	Long chain fatty acid	met-a-330
Oleate (18:1n9)	Known	Long chain fatty acid	met-a-332
Palmitate (16:0)	Known	Long chain fatty acid	met-a-329
Palmitoleate (16:1n7)	Known	Long chain fatty acid	met-a-576
Pentadecanoate (15:0)	Known	Long chain fatty acid	met-a-333
Stearate (18:0)	Known	Long chain fatty acid	met-a-331

Stearidonate (18:4n3)	Known	Long chain fatty acid	met-a-624
1-arachidonoylglycerophosphocholine	Known	Lysolipid	met-a-558
1-arachidonoylglycerophosphoethanolamine	Known	Lysolipid	met-a-682
1-arachidonoylglycerophosphoinositol	Known	Lysolipid	met-a-634
1-docosahexaenoylglycerophosphocholine	Known	Lysolipid	met-a-603
1-eicosadienoylglycerophosphocholine	Known	Lysolipid	met-a-607
1-eicosatrienoylglycerophosphocholine	Known	Lysolipid	met-a-602
1-heptadecanoylglycerophosphocholine	Known	Lysolipid	met-a-620
1-linoleoylglycerophosphocholine	Known	Lysolipid	met-a-655
1-linoleoylglycerophosphoethanolamine	Known	Lysolipid	met-a-497
1-myristoylglycerophosphocholine	Known	Lysolipid	met-a-705
1-oleoylglycerophosphocholine	Known	Lysolipid	met-a-621
1-oleoylglycerophosphoethanolamine	Known	Lysolipid	met-a-706
1-palmitoleoylglycerophosphocholine	Known	Lysolipid	met-a-559
1-palmitoylglycerophosphocholine	Known	Lysolipid	met-a-619
1-palmitoylglycerophosphoethanolamine	Known	Lysolipid	met-a-707
1-palmitoylglycerophosphoinositol	Known	Lysolipid	met-a-692
1-palmitoylplasmenylethanolamine	Known	Lysolipid	#N/A
1-stearoylglycerophosphocholine	Known	Lysolipid	met-a-622
1-stearoylglycerophosphoethanolamine	Known	Lysolipid	met-a-654
1-stearoylglycerophosphoinositol	Known	Lysolipid	met-a-410
2-linoleoylglycerophosphocholine	Known	Lysolipid	met-a-690
2-linoleoylglycerophosphoethanolamine	Known	Lysolipid	#N/A
2-oleoylglycerophosphocholine	Known	Lysolipid	met-a-688
2-palmitoylglycerophosphocholine	Known	Lysolipid	met-a-687
2-stearoylglycerophosphocholine	Known	Lysolipid	met-a-689

10-undecenoate (11:1n1)	Known	Medium chain fatty acid	met-a-482
5-dodecenoate (12:1n7)	Known	Medium chain fatty acid	met-a-623
Caprate (10:0)	Known	Medium chain fatty acid	#N/A
Caproate (6:0)	Known	Medium chain fatty acid	met-a-480
Caprylate (8:0)	Known	Medium chain fatty acid	met-a-481
Heptanoate (7:0)	Known	Medium chain fatty acid	met-a-349
Laurate (12:0)	Known	Medium chain fatty acid	met-a-350
Pelargonate (9:0)	Known	Medium chain fatty acid	met-a-366
Undecanoate (11:0)	Known	Medium chain fatty acid	met-a-367
1-linoleoylglycerol (1-monolinolein)	Known	Monoacylglycerol	met-a-445
1-oleoylglycerol (1-monoolein)	Known	Monoacylglycerol	met-a-424
1-palmitoylglycerol (1-monopalmitin)	Known	Monoacylglycerol	met-a-423
1-stearoylglycerol (1-monostearin)	Known	Monoacylglycerol	met-a-425
Valerate	Known	Short chain fatty acid	met-a-575
Palmitoyl sphingomyelin	Known	Sphingolipid	met-a-751
4-androsten-3beta,17beta-diol disulfate 1	Known	Sterol, Steroid	met-a-747
4-androsten-3beta,17beta-diol disulfate 2	Known	Sterol, Steroid	met-a-748
5alpha-androstan-3beta,17beta-diol disulfate	Known	Sterol, Steroid	met-a-746
5alpha-pregnan-3beta,20alpha-diol disulfate	Known	Sterol, Steroid	#N/A
7-alpha-hydroxy-3-oxo-4-cholestenoate (7-Hoca)	Known	Sterol, Steroid	met-a-737
Androsterone sulfate	Known	Sterol, Steroid	met-a-460
Cholesterol	Known	Sterol, Steroid	met-a-307
Cortisol	Known	Sterol, Steroid	met-a-353
Cortisone	Known	Sterol, Steroid	met-a-354
Dehydroisoandrosterone sulfate (DHEA-S)	Known	Sterol, Steroid	met-a-478
Epiandrosterone sulfate	Known	Sterol, Steroid	met-a-627

Estrone 3-sulfate	Known	Sterol, Steroid	met-a-403
Lathosterol	Known	Sterol, Steroid	met-a-579
X-11445--5-alpha-pregnan-3beta,20alpha-disulfate	Identified	Sterol/Steroid	met-a-517
X-12441--12-hydroxyeicosatetraenoate (12-HETE)	Identified	Eicosanoid	met-a-609
X-12442--5,8-tetradecadienoate	Identified	Long chain fatty acid	met-a-610
X-12990--docosapentaenoic acid (n6-DPA)	Identified	Essential fatty acid	met-a-671
X-13183--stearamide	Identified	Fatty acid amide	met-a-674
X-13431--nonanoylcarnitine	Identified	Carnitine metabolism	met-a-684

Table S2. Downstream molecules of eight lipid metabolites related to H-BMD. Downstream molecules of eight lipid metabolites related to H-BMD. We explored eight lipid metabolites related to H-BMD in the KEGG database and Wikipathy database, and only two of the eight had known metabolic pathways.(Myo-inositol: Phosphatidylinositol signaling system, Glycerophospholipid biosynthetic pathway and Inositol phosphate metabolism; Acetylcarnitine: Alanine and aspartate metabolism and insulin resistance. However, the downstream modules of Glycerophospholipid biological pathway did not find the corresponding gwas-id in the GWAS database). Among the downstream molecules of the obtained results, only a few have codes in the IEU OpenGWAS project. EC: Enzyme Commission number in KEGG； GWAS-ID: The corresponding ID of the downstream molecule in the IEU OpenGWAS project. URL of the IEU OpenGWAS project: https://gwas.mrcieu.ac.uk/ (accessed on 31 August 2023).

metabolite	pahtway	EC	Names of downstream molecules	Symbol	GWAS-ID
Myo-inositol	Phosphatidylinositol signaling system	3.1.3.36	inositol polyphosphate 5-phosphatase	INPP5B_F	prot-a-1557
		PTEN	phosphatidylinositol-3,4,5-trisphosphate 3-phosphatase and dual-specificity protein phosphatase	PTEN	prot-a-2425
		PIK3C	phosphatidylinositol-4,5-bisphosphate 3-kinase catalytic subunit	PIK3CA_B_D	prot-a-2270
		3.1.3.95	myotubularin	MTM1	prot-a-1963

		3.1.3.64	myotubularin	MTM1	prot-a-1963
		3.1.3.56	inositol polyphosphate 5-phosphatase	INPP5A	prot-a-1557
		CALM	calmodulin	CALM	prot-a-2218
		2.7.1.107	diacylglycerol kinase	DGKA	prot-a-811
Myo-inositol	Inositol phosphate metabolism	3.1.3.36	inositol polyphosphate 5-phosphatase	OCRL	prot-a-2141
		3.1.3.56	inositol polyphosphate 5-phosphatase	I5P1	prot-a-1557
		3.1.3.64	myotubularin	MTM1	prot-a-1963
		3.1.3.67	phosphatidylinositol-3,4,5-trisphosphate 3-phosphatase and dual-specificity protein phosphatase	PTEN	prot-a-2425
		3.1.3.95	myotubularin	MTM1	prot-a-1963
		2.7.1.153	phosphatidylinositol-4,5-bisphosphate 3-kinase catalytic subunit alpha/beta/delta	PIK3CA/B/D	prot-a-2270
		3.1.4.11	phosphatidylinositol phospholipase C, gamma-1	PLCG1	prot-c-4563_61_2

		5.3.1.1	triosephosphate isomerase	TIPS	prot-a-3083
Acetylcarnitine	Alanine and aspartate metabolism	6.3.1.1	aspartate—ammonia ligase	asnA	prot-a-190
		4.3.2.2	adenylosuccinate lyase	PUR8	prot-c-5023_23_1
Acetylcarnitine	Insulin resistance	INSR	insulin receptor	INSR	prot-a-1564
		3.1.3.36	inositol polyphosphate 5-phosphatase	INPP5B_F	prot-a-1557
		PTEN	phosphatidylinositol-3,4,5-trisphosphate 3-phosphatase and dual-specificity protein phosphatase	PTEN	prot-a-2425
		PIK3C	phosphatidylinositol-4,5-bisphosphate 3-kinase catalytic subunit	PIK3CA_B_D	prot-a-2270
		3.1.3.95	myotubularin	MTM1	prot-a-1963
